# Odontalgia

**Published:** 1906-12-15

**Authors:** Wm. D. McFadden

**Affiliations:** Lockport, New York


### ODONTALGIA.

BY WM. D. MCFADDEN, D.D.S., LOCKPORT, NEW YORK.

*Pulpitis—due to caries*—With a water syringe wash the cavity thoroughly with a warm aqueous solution of sodium bicarbonate, to neutralize any acid present and to remove any food particles causing irritation: then apply rubber dam and dry the cavity thoroughly with cotton, after which place in the cavity one Parke, Davis & Co., hypodermic tablet No, 151. I dissolve this in a minim or two of their 1-1000 Solution Adrenälin Chloride as it is non-irritating. Place a rubber plug over the cavity opening and make slight pressure but not enough to irritate. If the pulp is exposed continue the treatment until it is anaesthetized: extirpation will then be painless.

In case the pulp is not exposed and extirpation is contraindicated, use No. 151 tablet, as before; then place in the cavity cotton saturated with Dentalone (warm) and seal it into the cavity. It is very important that the cavity be washed and dried thoroughly, as above, before treatment, and of course it is necessary to use all antiseptic precautions.

*Pericementitis after Death of the P.ulp.*—Wash the cavity to remove all debris, place rubber dam over the crown, clamping on the adjacent tooth if possible-

Carefully remove all debris from the canals with a sterile broach. This must be done most thoroughly, using all antiseptic precautions. Place in the cavity one No. 151 tablet P. D. & Co., and dissolve with 1-1000 Andrealin Solution.

Then force the smooth broach carefully down the canal through the apex of the root. In this manner I have been successful in relieving many of the most painful cases in a few minutes. After the pain ceases I carefully pump into the canals a solution of Chloretone in oil of cloves, or Dentalone, which is both antiseptic and obtundent. Cotton is placed loosely in the cavity and on the following day, if the pericementitis has subsided, I begin antiseptic treatment preparatory to filling the root canals.

